# Isolation of Peripheral Blood Mononuclear Cells from Macaques on a Density Barrier

**DOI:** 10.1100/tsw.2002.845

**Published:** 2002-06-15

**Authors:** John Graham

**Affiliations:** School of Biomolecular Sciences, Liverpool John Moores University, UK

**Keywords:** Peripheral blood mononuclear cells, Macaques, OptiPrep^TM^, iodixanol, density barrier

## Abstract

The standard techniques for the isolation of human peripheral blood mononuclear cells (PBMCs) using commercial “lymphocyte isolation media” cannot be satisfactorily extended to experimental animals without manipulating either the density or the osmolality of the medium. PBMCs from Macaques can also be isolated from whole blood by sedimentation on to a density barrier containing approx. 10% iodixanol, polysucrose (Ficoll) with a density of approx 1.074 g/ml.

**METHOD TYPE:** extraction, isolation, purification and separation

**SUB METHOD TYPE:** centrifugation

## INTRODUCTION

For the isolation of human peripheral blood mononuclear cells (PBMCs) from whole blood, sedimentation onto a barrier of density 1.077 g/ml is probably the most widely used technique. A variety of commercial medium containing diatrizaote and a polysaccharide are available. Nycoprep™ 1.077, which contains a buffered solution of Nycodenz®, or a medium of the same density prepared from OptiPrep™[1] are alternatives which contain a nonionic gradient solute and avoid the use of polysaccharide. Nycoprep™ 1.077A is designed for blood from experimental animals (rodents and rabbits).

None of these density barrier media are totally successful for the isolation of PBMCs from the blood of other primates (e.g., Macaques). The method described in this Protocol Article using a density barrier of approx. 1.074 g/ml containing iodixanol and a polysaccharide was devised by Stittelaar et al.[2]. For another possible strategy see Note 1.

## MATERIALS AND EQUIPMENT

OptiPrep™ (shake gently before use)
Ten-times concentrated phosphate buffered saline (10xPBS)
Polysaccharide solution: 6% (w/v) polysucrose 400 (MWt 400,000) in water

Plastic conical centrifuge tubes (12–15 ml)
Syringe with metal cannula or plastic Pasteur pipette
Low-speed (temperature-controlled) centrifuge with swinging-bucket rotor

## METHOD

1. Prepare the density barrier by mixing OptiPrep™, 10xPBS and the 60% polysucrose in the following volume ratio: 16.7**:**8.3**:**75.
2. In a centrifuge tube layer 2 vol of heparinized blood over 1 vol of the density barrier (see Note 2).
3. Centrifuge at 600*g* for 20 min.
4. Remove the PBMC that band at the interface.
5. Dilute the collected material with 2 vol of buffered-saline and pellet the cells at 250–500*g* for 5–10 min.

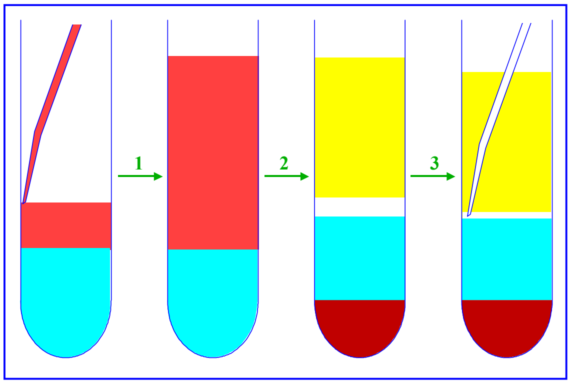

**FIGURE 1**. Isolation of Macaque PBMCs on a density barrier. (1) 2 vol of blood layered over 1 vol of medium; (2-3) after centrifugation, the PBMC band is harvested by aspiration.

## NOTES

1. An alternative strategy to the sedimentation of PBMCs onto a density barrier is to adjust the density of the plasma of whole blood to approx. 1.078 g/ml and then allow these cells to float to the surface during the centrifugation[3]. Details of this technique as applied to human, rat, mouse, and bovine blood are given in Ref. [4]. This flotation approach seems more widely applicable to various species and it may be worth investigating for blood from primates other than man.

2. The blood might be applied to the density barrier after dilution with an equal volume of PBS (as is recommended for human blood) rather than undiluted; use whichever method provides the optimal results.

## ACKNOWLEDGEMENTS

The author and TheScientificWorld wish to thank Axis-Shield PoC, AS, Oslo, Norway for their kind permission to adapt OptiPrep™ Application Sheet C30 in the preparation of this Protocol Article.
